# Switching from Vitamin K Antagonists to Direct Oral Anticoagulants in Frail Elderly Patients with Atrial Fibrillation: Systematic Review and Meta-Analysis

**DOI:** 10.3390/medsci14050516

**Published:** 2026-08-25

**Authors:** Paschalis Karakasis, Ricardo Gómez-Huelgas, Stylianos Tzeis, Konstantinos Vlachos, Konstantinos C. Siontis, Alberto M. Marra, Konstantinos Pamporis, Antonios P. Antoniadis, Eleftheria Lefkou, Valentin A. Kokorin, Dimitrios Patoulias, Nikolaos Fragakis

**Affiliations:** 1 Second Department of Cardiology, Aristotle University of Thessaloniki, Hippokration General Hospital, 546 42 Thessaloniki, Greece; 2 Internal Medicine Department, Regional University Hospital of Málaga, Biomedical Research Institute of Málaga (IBIMA), University of Málaga (UMA), 29010 Malaga, Spain; ricardogomezhuelgas@hotmail.com; 3 Department of Cardiology, Mitera Hospital, 6 Erythrou Stavrou Str., 151 23 Marousi, Greece; stzeis@otenet.gr; 4 Electrophysiology and Heart Modeling Institute, IHU Liryc, Fondation Bordeaux Université, Université de Bordeaux, INSERM U1045, 33604 Pessac, France; vlachos.konstantinos7@gmail.com; 5 Cardiac Arrhythmia Department, INSERM U1045, CHU de Bordeaux, 33604 Pessac, France; 6 Electrophysiology Department, Hygeia Hospital, 151 23 Athens, Greece; 7 Department of Cardiovascular Medicine, Mayo Clinic, Rochester, MN 55905, USA; 8 Department of Translational Medical Sciences, “Federico II” University, 80131 Naples, Italy; 9 Department of Internal Medicine and Clinical Complexity, “Federico II” University Hospital, 80131 Naples, Italy; 10 Interdepartmental Center for Gender Medicine Research “GENESIS”, Federico II University, 80131 Naples, Italy; 11 First Department of Cardiology, School of Medicine, National and Kapodistrian University of Athens, Hippokration General Hospital, 115 27 Athens, Greece; 12 Hematology-Transfusion Medicine Department, Faculty of Medicine, Larissa University Hospital, University of Thessaly, 413 34 Larissa, Greece; 13 Department of Hospital Therapy with Courses in Endocrinology, Hematology and Clinical Laboratory Diagnostics, Peoples’ Friendship University of Russia Named after Patrice Lumumba (RUDN University), Moscow 117198, Russia; valentinkokorin@yahoo.com; 14 Department of Hospital Therapy Named after Academician P.E. Lukomsky, Pirogov Russian National Research Medical University, Moscow 117513, Russia; 15 Second Propedeutic Department of Internal Medicine, Faculty of Medicine, School of Health Sciences Aristotle, University of Thessaloniki, 541 24 Thessaloniki, Greece

**Keywords:** atrial fibrillation, frailty, elderly, vitamin K antagonists, warfarin, direct oral anticoagulants, switching, anticoagulation, major bleeding, thromboembolism, mortality

## Abstract

**Background**: Frailty complicates anticoagulation management in older patients with atrial fibrillation (AF), particularly when long-term vitamin K antagonist (VKA) therapy is already established. Whether switching to a direct oral anticoagulant (DOAC) improves outcomes in this setting remains uncertain, and guideline recommendations are limited. We performed a systematic review and meta-analysis comparing outcomes of VKA-to-DOAC switching versus VKA continuation in frail elderly patients with AF. **Methods**: MEDLINE, Scopus, and the Cochrane Library were systematically searched from inception through 4 April 2026. Studies enrolling frail elderly patients with AF receiving VKA therapy and directly comparing switching to a DOAC versus continued VKA treatment were eligible. The primary outcome was a composite of stroke/systemic embolism, major bleeding, or all-cause mortality. Adjusted hazard ratios (HRs) were pooled using three-level random-effects models. **Results:** Four studies (five treatment comparisons) comprising 57,099 patients were included. Overall, switching from VKA to DOAC therapy was not associated with a significant difference in the composite outcome compared with continued VKA treatment (HR 1.18, 95% CI 0.84–1.67). No significant differences were observed for major bleeding (HR 1.13, 95% CI 0.80–1.60), thromboembolism (HR 1.03, 95% CI 0.63–1.68), or all-cause mortality (HR 0.95, 95% CI 0.77–1.16). In analyses restricted to randomized trials, results remained neutral across all outcomes. **Conclusion**: In frail elderly patients with AF receiving VKA therapy, switching to a DOAC was not associated with significant reductions in thromboembolism, major bleeding, mortality, or the composite outcome compared with continued VKA treatment.

## 1. Introduction

Atrial fibrillation (AF) is the most common sustained cardiac arrhythmia and a major cause of stroke, heart failure, hospitalization, cognitive decline, and death, particularly in older adults [1]. Oral anticoagulation is central to AF management, and current guidelines generally favor direct oral anticoagulants (DOACs) over vitamin K antagonists (VKAs) for most patients with non-valvular AF because of their lower risk of intracranial bleeding and at least comparable efficacy for stroke and systemic embolism prevention [2,3,4]. However, this general preference does not fully address a distinct clinical scenario: the frail elderly patient who is already established on long-term VKA therapy.

Frailty complicates anticoagulation decisions because it is associated with reduced physiologic reserve, multimorbidity, polypharmacy, and increased susceptibility to both thromboembolic and bleeding events [5,6]. Frail older adults were also underrepresented in the pivotal DOAC trials, limiting direct extrapolation of their findings to this population [7]. In patients already receiving VKA therapy, the relevant clinical question is therefore not whether anticoagulation should be initiated, but whether transition from an established VKA regimen to a DOAC provides additional clinical benefit [8,9].

Evidence addressing this question remains limited and inconsistent. The 2024 ESC guidelines acknowledge this uncertainty and indicate that continuation of VKA therapy may be considered in clinically stable older patients with good anticoagulation control, whereas the 2023 ACC/AHA/ACCP/HRS guidelines provide no specific recommendation for VKA-to-DOAC switching in frail elderly patients with AF [2,3]. Available randomized and observational studies have produced discordant findings [10,11,12], and these data have not yet been comprehensively synthesized.

Accordingly, the present systematic review and meta-analysis aimed to compare clinical outcomes associated with switching from VKA to DOAC therapy versus continuing VKA treatment in frail elderly patients with AF.

## 2. Material and Methods

This investigation was conducted in accordance with the methodological standards set forth in the Cochrane Handbook for Systematic Reviews [13] and was reported in line with the Preferred Reporting Items for Systematic Reviews and Meta-Analyses (PRISMA) 2020 statement [14]. The protocol was prospectively registered in the Open Science Framework (https://doi.org/10.17605/OSF.IO/SP8JD) and was followed as prespecified, with no departures from the original methodological plan. Because the study was based solely on aggregate data extracted from previously published reports, neither institutional ethics approval nor informed consent was required.

### 2.1. Search Strategy

The search strategy was developed collaboratively by three investigators, followed by a comprehensive and independent literature search conducted across MEDLINE (via PubMed), Scopus, and the Cochrane Database of Systematic Reviews from database inception through 4 April 2026. No restrictions were imposed on language, publication year, publication status, or date of dissemination. Search syntaxes were constructed using combinations of free-text terms and Medical Subject Headings (MeSH) to capture the key concepts of vitamin K antagonists, warfarin, acenocoumarol, direct oral anticoagulants, and atrial fibrillation. To enhance retrieval sensitivity and minimize the risk of missing relevant studies, supplementary searches were also performed in ClinicalTrials.gov, Epistemonikos, and Google Scholar. In addition, backward and forward citation tracking was undertaken using the citationchaser R package [15], further strengthening the comprehensiveness of study identification. The full electronic search strategies are provided in Supplemental Tables S1–S3.

### 2.2. Eligibility Criteria

#### 2.2.1. Inclusion Criteria

Eligible studies were those enrolling frail elderly patients with AF who were receiving VKA therapy and directly compared switching to a DOAC with continued VKA treatment. Both randomized and observational comparative studies were considered eligible, provided that they reported at least one outcome of interest and included sufficient data for quantitative synthesis. Frailty could be defined according to an established frailty measure, administrative frailty index, claims-based frailty construct, or an equivalent operational definition used by the primary investigators.

#### 2.2.2. Exclusion Criteria

The following study designs and publication types were deemed ineligible for inclusion: (i) descriptive reports without a comparative analytic framework, including case reports and case series, as well as narrative reviews; (ii) non-original or interpretive publications, such as editorials, letters, commentaries, and expert opinion articles; (iii) non-full-text or non-peer-reviewed sources, including conference abstracts, study protocols, clinical practice guidelines, and unpublished academic theses or dissertations; and (iv) observational or interventional designs not aligned with the prespecified eligibility framework, specifically cross-sectional studies, case–control studies, and crossover trials.

### 2.3. Outcomes

The prespecified primary outcome was a composite of stroke/systemic embolism, major bleeding, or all-cause mortality. This endpoint was adopted from the composite outcomes reported in the primary studies and was selected to preserve conceptual consistency while providing an integrated assessment of effectiveness, safety, and survival.

Secondary outcomes included major bleeding, thromboembolic events, and all-cause mortality examined as individual endpoints. Thromboembolic events were defined in accordance with each study’s reporting structure and typically comprised ischemic stroke, systemic embolism, or their composite. Major bleeding was recorded using the definitions applied by the original investigators. Where endpoint definitions differed across studies, outcomes were synthesized under the same prespecified clinical construct on the basis of the original study-level definitions. All-cause mortality referred to death from any cause during follow-up.

### 2.4. Study Selection

Study selection was performed in a two-stage process by three investigators working independently. In the first stage, all records retrieved through the prespecified search strategy underwent title and abstract screening. To preserve sensitivity and minimize the risk of erroneous early exclusion, any record considered potentially relevant by at least one reviewer was advanced to full-text assessment. In the second stage, the same reviewers independently examined the full-text reports against the predefined eligibility criteria to establish final study inclusion. Any discrepancies were resolved through discussion and consensus, with adjudication by a senior author where required. Screening was carried out using the Abstrackr platform [16], whereas citation organization and duplicate management were undertaken in Mendeley.

### 2.5. Data Extraction

A standardized data extraction form was developed a priori and refined through pilot testing to optimize completeness, consistency, and interpretability before formal extraction commenced. After reviewer calibration, the finalized form was applied uniformly to all eligible studies. Data extraction was undertaken independently by three investigators, with disagreements resolved by consensus and, when required, adjudication by a senior author.

Extracted data were organized into two prespecified domains. The first encompassed study-level characteristics, including author, year of publication, country, study design, data source, enrollment period, sample size, follow-up duration, frailty definition or operational frailty construct, criteria used to define the elderly population, exposure ascertainment, comparator definition, and reported outcome measures.

The second domain comprised baseline population characteristics overall and by treatment group, including age, sex, and clinically relevant comorbidities and risk features, such as heart failure, hypertension, diabetes mellitus, prior thromboembolism, prior bleeding, renal dysfunction, and other variables pertinent to thromboembolic and hemorrhagic risk. Outcome-level data were extracted in parallel, including endpoint definitions, adjusted effect estimates, corresponding 95% confidence intervals, and raw event counts where available.

When multiple models were reported, the most fully adjusted estimates were preferentially selected for quantitative synthesis. Where essential data were incomplete or insufficiently detailed, attempts were made to obtain additional information from the corresponding authors of the original studies.

### 2.6. Quality Assessment

Risk of bias was evaluated independently by three investigators according to study design. Randomized trials were appraised using the revised Cochrane Risk of Bias 2 (RoB 2) instrument [17], whereas non-randomized studies were assessed with the Risk Of Bias In Non-randomized Studies of Interventions (ROBINS-I) tool [18]. Any discordance in domain-level judgments or overall ratings was resolved through reviewer discussion and consensus, with final adjudication by a senior reviewer when necessary.

### 2.7. Data Analysis

All statistical analyses were performed in R (version 4.2.0). The principal summary measure was the HR with corresponding 95% confidence intervals (CIs). For each eligible study, the most fully adjusted HR estimates were preferentially extracted whenever available to preserve the effect measures reported by the primary investigations and to maximize comparability across studies. When adjusted HRs were not directly reported, as in the study by Kim et al., time-to-event effect estimates were derived from available binomial data using established conversion methods [19] that have been applied and validated in prior meta-analyses from our group [20]. This approach approximates the log-HR from the reported event proportions and relies principally on the assumptions of proportional hazards and limited censoring at the time point at which the binomial outcome is assessed, with the variance of the log-HR approximated using a Taylor-series expansion. These converted estimates were subsequently incorporated into the primary pooled analysis.

Summary hazard ratios (HRs) were calculated using three-level random-effects models to account for anticipated between-study clinical and methodological heterogeneity. Because Kim et al. contributed two agent-specific comparisons (apixaban versus continued warfarin and rivaroxaban versus continued warfarin), these estimates were assigned to the same study cluster in the three-level random-effects model. The model was implemented using the meta package in R, which internally uses rma.mv from the metafor package for clustered effect estimates. Variance components were estimated using restricted maximum likelihood. The default within-cluster sampling correlation (rho = 0) was retained because the covariance between the two agent-specific estimates was not available from the primary study. Between-study variance was estimated under a frequentist framework using restricted maximum likelihood. Statistical significance was defined by a two-sided *p* value < 0.05.

Statistical heterogeneity was quantified with the I^2^ statistic and assessed formally using Cochran’s Q test. In keeping with conventional thresholds, I^2^ values of 0–30% were interpreted as low heterogeneity, 30–50% as moderate, 50–75% as substantial, and 75–100% as considerable heterogeneity [21]. Given the limited number of included studies and the substantial between-study heterogeneity, prediction intervals were not calculated because they would be expected to be insufficiently informative for clinical interpretation.

The robustness of the primary findings was examined in a prespecified sensitivity analysis in which the dataset was restricted to randomized controlled trials (RCTs) only, to assess the consistency of the pooled estimates within a more rigorous study-design framework. Because comparable outcome estimates for individual DOAC agents were not consistently available across the included studies, agent-specific meta-analyses were not feasible; therefore, the primary pooled estimates represent a class-level effect of switching from VKA to DOAC therapy.

As fewer than 10 studies were included, formal evaluation of small-study effects and publication bias was not considered reliable and was therefore not performed.

### 2.8. Certainty of Evidence

The certainty of evidence for each prespecified outcome was evaluated using the Grading of Recommendations Assessment, Development and Evaluation (GRADE) framework. Certainty was assessed across the domains of risk of bias, inconsistency, indirectness, imprecision, and publication bias and categorized as high, moderate, low, or very low.

## 3. Results

### 3.1. Study Selection and Characteristics

**Figure 1:**
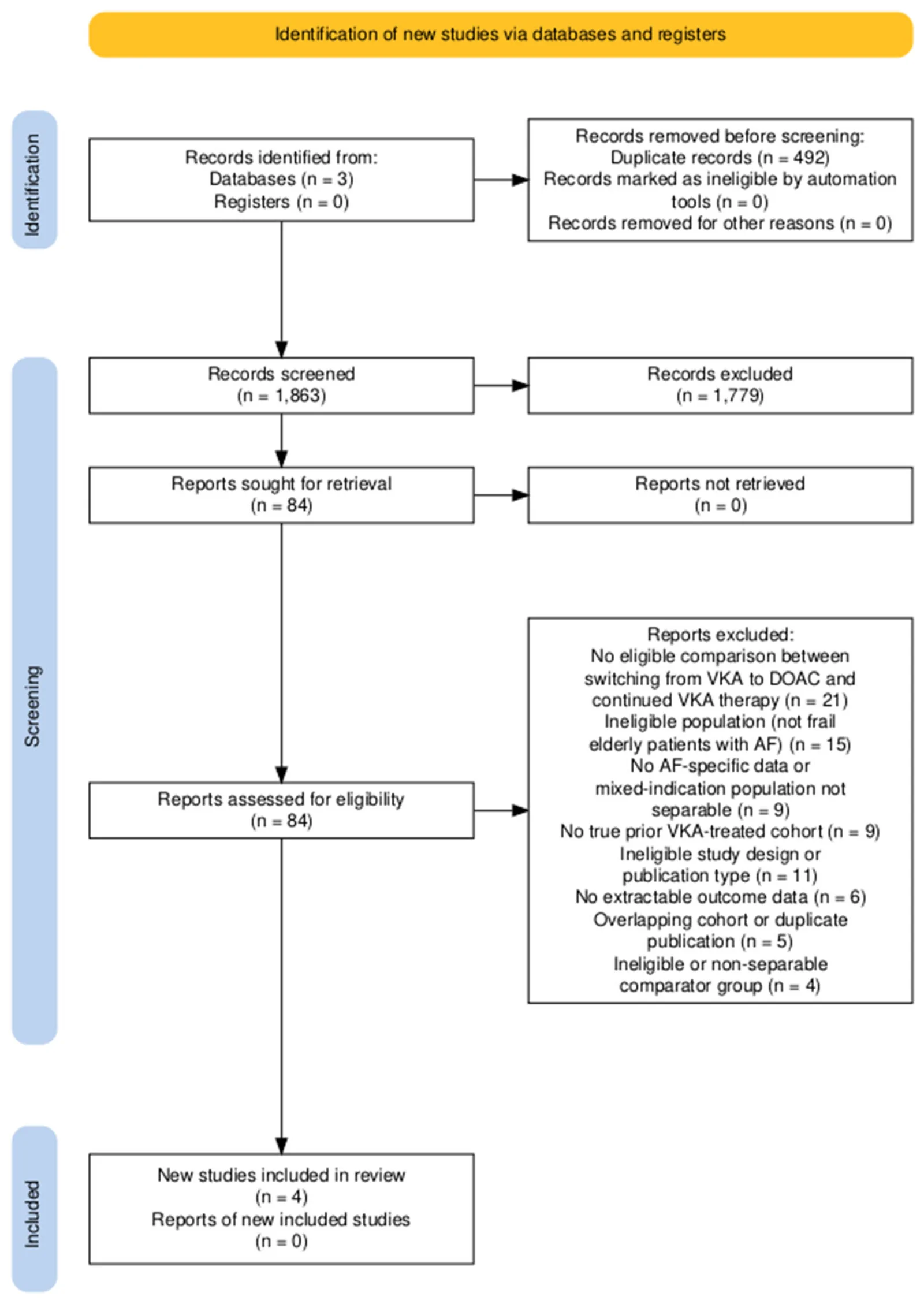
PRISMA 2020 flow diagram of study selection.

The study selection process is detailed in the PRISMA 2020 flow diagram (Figure 1). A total of 1863 records were screened after database searching, while 492 duplicate records were identified and removed prior to screening. Following title and abstract review, 1779 records were excluded. The remaining 84 reports were retrieved for full-text evaluation, all of which were successfully assessed for eligibility. After full-text review, 80 reports were excluded for prespecified reasons, and 4 studies were ultimately included in the systematic review and meta-analysis [10,11,12,22].

The main characteristics of the included studies are summarized in Table 1. A total of 4 studies, contributing 5 treatment comparisons, were included, comprising 57,099 patients with AF. Of these, 25,717 patients received a DOAC strategy after warfarin exposure or switching, whereas 31,382 continued VKA therapy. The evidence base consisted of 2 RCTs and 2 nationwide observational cohort studies, including propensity score–matched and time-varying exposure analyses. Follow-up ranged from 12 to 26.7 months. Mean age ranged from 79.3 to 83.0 years. The proportion of women ranged from 36.2% to 54.7%. Baseline CHA_2_DS_2_-VASc values ranged from 3.9 to 5.1. Comorbidity burden was substantial across the included cohorts. Heart failure prevalence ranged from 11.7% to 52.0%, hypertension from 50.8% to 93.4%, and diabetes from 21.1% to 37.8%.

The risk-of-bias assessments for the included randomized and non-randomized studies are presented in Supplemental Figure S1. Both randomized studies were judged as having some concerns overall under RoB 2, primarily related to deviations from intended interventions and/or selection of the reported result. Both observational studies were judged as having a moderate overall risk of bias under ROBINS-I, mainly because of concerns regarding confounding, participant selection, and selected study-specific domains. No study was judged to have a high, serious, or critical overall risk of bias.

### 3.2. Main Analysis

Five treatment comparisons from four studies were included in the meta-analysis for each outcome, representing 57,099 participants overall, including 25,717 in the DOAC group and 31,382 in the VKA group.

**Figure 2:**
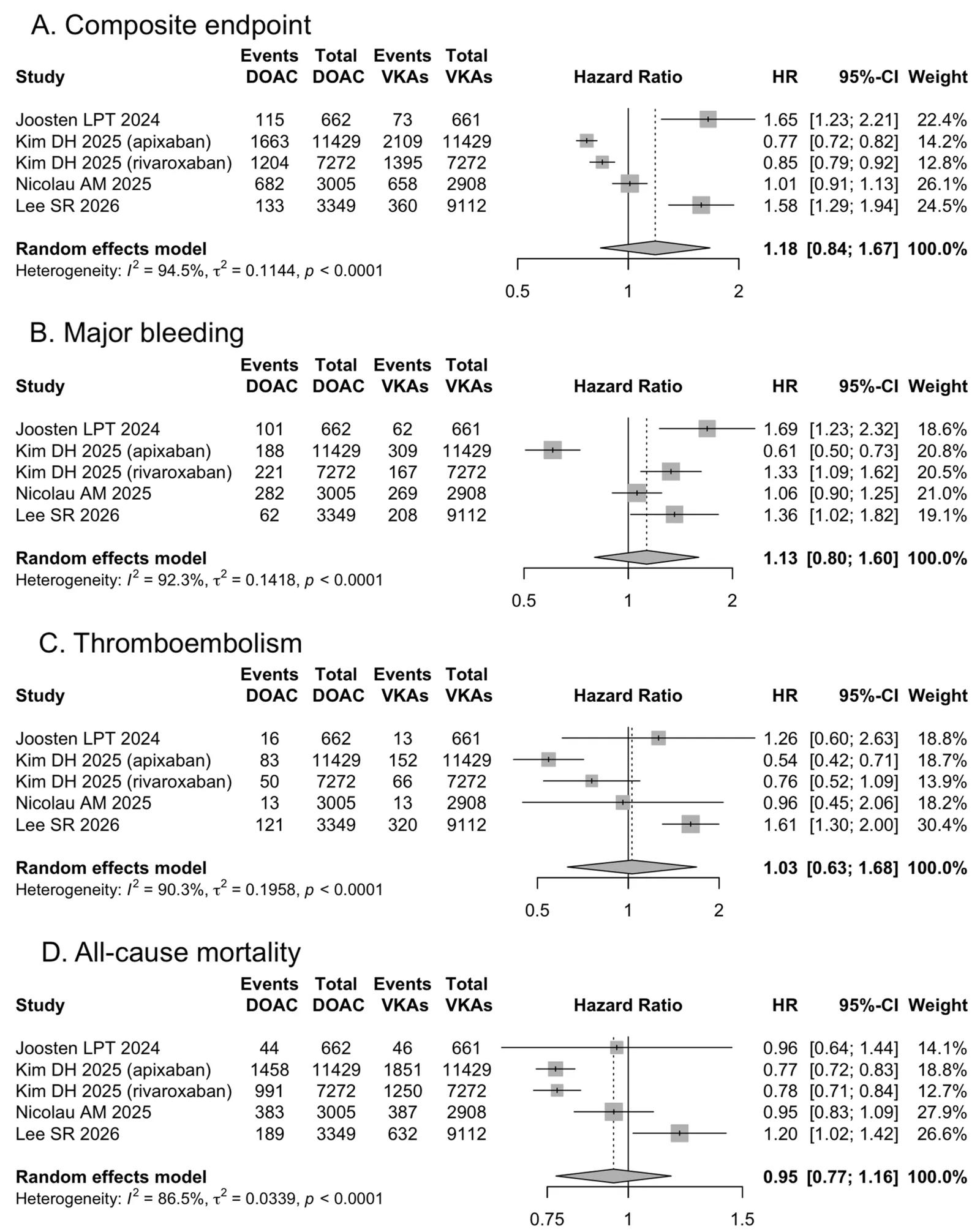
Forest plots depicting individual and pooled HRs with 95% CIs for the comparison between switching from VKA therapy to DOAC treatment and remaining on VKA therapy for (**A**) the composite endpoint of stroke/systemic embolism, major bleeding, or all-cause mortality; (**B**) major bleeding; (**C**) thromboembolism; and (**D**) all-cause mortality. Square markers denote study-specific effect estimates weighted according to their contribution to the meta-analysis, and horizontal lines represent 95% CIs. Diamonds indicate pooled random-effects estimates. Values below 1.0 favor DOAC switching, whereas values above 1.0 favor continued VKA therapy. Abbreviations: AF, atrial fibrillation; CI, confidence interval; DOAC, direct oral anticoagulant; HR, hazard ratio; SEE, systemic embolic event; VKA, vitamin K antagonist [11,12,13,23].

For the composite endpoint of stroke/systemic embolism, major bleeding, or all-cause mortality, 8212 events were recorded overall, with 3617 events in the DOAC group and 4595 in the VKA group. There was no significant difference between treatment strategies (HR 1.18, 95% CI 0.84–1.67, *p* = 0.33; I^2^ = 94.5%, Figure 2A).

For major bleeding, a total of 1869 events occurred, including 854 in the DOAC group and 1015 in the VKA group. The pooled analysis showed no significant difference in major bleeding risk between patients who switched to a DOAC and those who remained on VKA therapy (HR 1.13, 95% CI 0.80–1.60, *p* = 0.49; I^2^ = 92.3%, Figure 2B).

For thromboembolism, 847 events were observed overall, of which 283 occurred in the DOAC group and 564 in the VKA group. The risk of thromboembolic events was comparable between the two treatment strategies (HR 1.03, 95% CI 0.63–1.68, *p* = 0.91; I^2^ = 90.3%, Figure 2C).

For all-cause mortality, 7231 deaths were reported, including 3065 in the DOAC group and 4166 in the VKA group. All-cause mortality did not differ significantly between patients who switched to DOAC therapy and those who continued VKA treatment (HR 0.95, 95% CI 0.77–1.16, *p* = 0.62; I^2^ = 86.5%, Figure 2D).

### 3.3. Sensitivity Analysis Restricted to RCTs

In the sensitivity analysis restricted to randomized trials, two study comparisons were included, comprising 7236 participants overall, with 3667 assigned to the DOAC group and 3569 to the VKA group.

**Figure 3:**
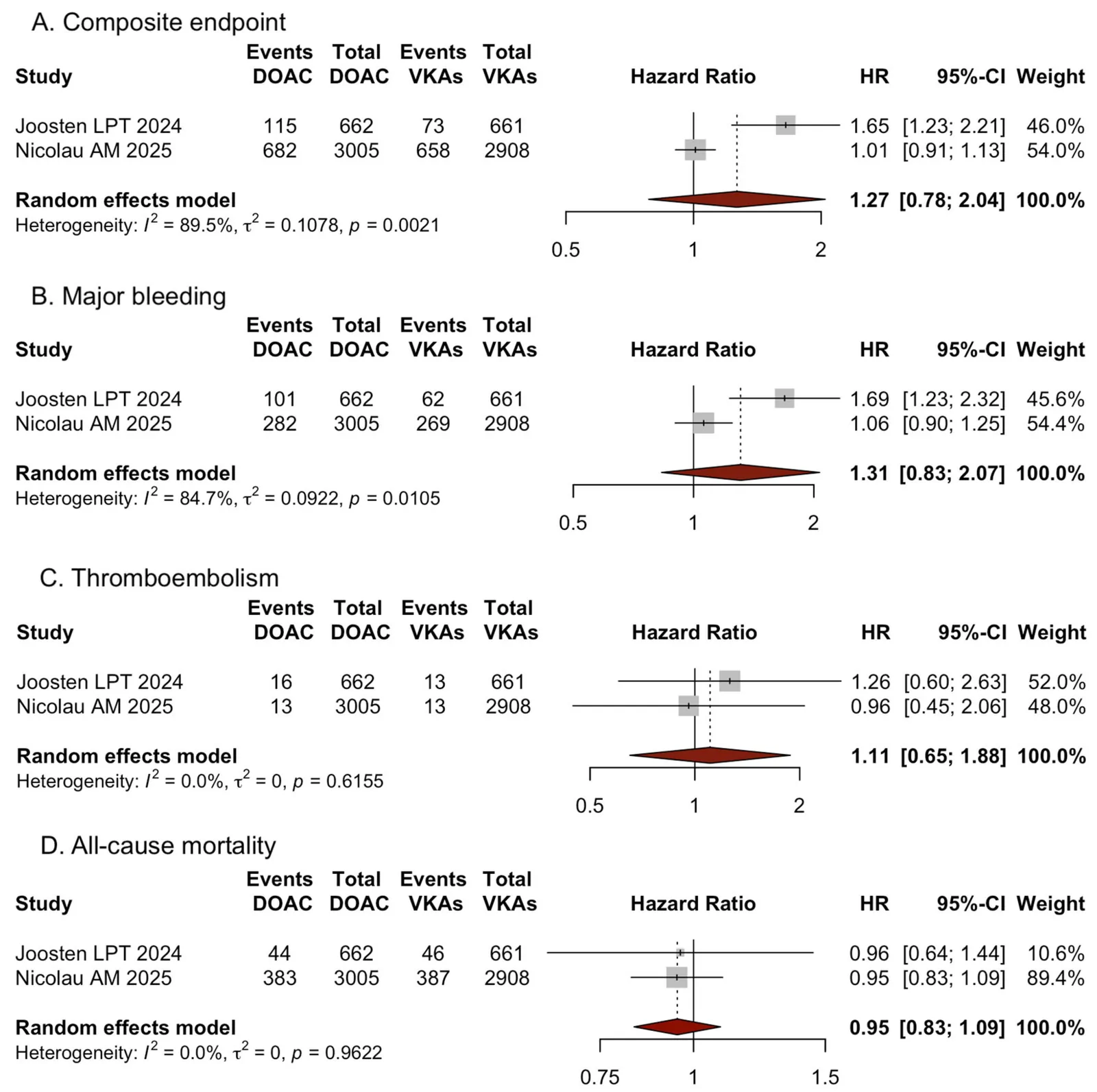
Sensitivity analysis restricted to randomized trials. Forest plots showing pooled HRs and 95% CIs for the comparison between switching from VKA therapy to DOAC treatment and continued VKA therapy in randomized trials only for (**A**) the composite endpoint of stroke/systemic embolism, major bleeding, or all-cause mortality; (**B**) major bleeding; (**C**) thromboembolism; and (**D**) all-cause mortality. Abbreviations: CI, confidence interval; DOAC, direct oral anticoagulant; HR, hazard ratio; VKA, vitamin K antagonist [10,22].

For the composite endpoint of stroke/systemic embolism, major bleeding, or all-cause mortality, a total of 1528 events were recorded, including 797 in the DOAC group and 731 in the VKA group. The pooled analysis showed no significant difference between treatment strategies (HR 1.27, 95% CI 0.78–2.04, *p* = 0.34; I^2^ = 89.5%, Figure 3A).

For major bleeding, 714 events were observed overall, with 383 events in the DOAC group and 331 in the VKA group. The risk of major bleeding did not differ significantly between patients switched to DOAC therapy and those who remained on VKA treatment (HR 1.31, 95% CI 0.83–2.07, *p* = 0.24; I^2^ = 84.7%, Figure 3B).

For thromboembolism, 55 events occurred overall, including 29 in the DOAC group and 26 in the VKA group. The pooled estimate showed no significant difference between the two treatment strategies (HR 1.11, 95% CI 0.65–1.88, *p* = 0.71; I^2^ = 0.0%, Figure 3C).

For all-cause mortality, 860 deaths were reported, of which 427 occurred in the DOAC group and 433 in the VKA group. No significant difference in all-cause mortality was observed between groups (HR 0.95, 95% CI 0.83–1.09, *p* = 0.46; I^2^ = 0.0%, Figure 3D).

### 3.4. Certainty of Evidence

The certainty of evidence was rated as low for all four outcomes. This rating was driven by serious inconsistency, reflecting substantial between-study heterogeneity and variability in effect estimates, and serious imprecision, as the confidence intervals encompassed potentially clinically important benefit and harm. The GRADE assessment for each outcome, together with the corresponding rationale for downgrading, is summarized in Table 2.

## 4. Discussion

This meta-analysis assembles the totality of available evidence to date—four studies (five treatment comparisons) and 57,099 patients—evaluating the clinical consequences of switching from VKA to DOAC therapy versus continued VKA treatment in frail elderly patients with AF. Three main findings emerge: (i) switching from VKA to DOAC therapy was not associated with a significant difference in the composite outcome of stroke/systemic embolism, major bleeding, or all-cause mortality compared with continued VKA treatment; (ii) no significant differences were observed for the individual endpoints of major bleeding, thromboembolism, or all-cause mortality; and (iii) these neutral findings remained consistent in the sensitivity analysis restricted to RCTs only, although the limited number of studies and wide confidence intervals warrant cautious interpretation and preclude conclusions of equivalence between treatment strategies.

Frailty and polypharmacy may substantially modify the balance between ischemic protection and bleeding hazard after anticoagulant transition. In ARISTOTLE, increasing concomitant medication burden was associated with higher rates of death, stroke/systemic embolism, and major bleeding, and the major bleeding advantage of apixaban over warfarin became progressively smaller with greater polypharmacy, suggesting that treatment complexity can attenuate the safety margin typically attributed to DOAC therapy [23]. Similarly, in ROCKET-AF, increasing numbers of background medications were linked to higher bleeding risk, whereas the relative efficacy of rivaroxaban versus warfarin remained broadly stable, indicating that polypharmacy may preferentially erode safety rather than thromboembolic protection [24]. Frailty appears to exert a comparable effect. In a dedicated ENGAGE AF-TIMI 48 frailty analysis, event rates for stroke, bleeding, and death rose progressively across the frailty spectrum, and although edoxaban remained broadly comparable to warfarin for efficacy, its bleeding advantage was no longer clearly evident in patients with severe frailty [25].

In the broader AF population, the literature on switching from VKA to DOAC therapy has been considerably more heterogeneous than the literature on initiating DOACs [26]. In studies evaluating switching to dabigatran, reported associations ranged from negligible effects to marked increases in ischemic stroke risk, with hazard ratios spanning from 1.10 to 5.79, while bleeding outcomes were similarly inconsistent, varying from modest risk reduction to substantial excess risk [27,28,29,30,31,32,33,34,35]. Evidence regarding rivaroxaban has also been mixed, with estimates for ischemic stroke ranging from modestly protective to modestly harmful and bleeding outcomes generally suggesting either little difference or a moderate increase in risk [27,29,30,31,32]. Notably, one case-crossover analysis identified a transient increase in the risk of ischemic stroke or systemic embolism, as well as any bleeding, within the first 60 days after treatment transition [27]. Interpretation of this literature, however, is constrained by important methodological limitations across studies, including insufficient adjustment for confounding [28,34,35], use of prevalent-user designs [31], vulnerability to immortal time bias [31], and, in several instances, limited statistical power to detect clinically meaningful differences [27,28,30,31,32].

Contemporary registry data suggest that switching from warfarin to DOACs is influenced not only by thromboembolic risk, but also by bleeding history, practice variation, and local prescribing patterns, indicating that treatment transition is a clinically selected process rather than a neutral therapeutic exchange [36]. This distinction is important, because the favorable class effect of DOACs versus warfarin demonstrated in pivotal trials and patient-level meta-analysis was derived largely from initiation cohorts and broad randomized populations, not from frail, VKA-experienced patients who had already demonstrated treatment persistence on long-term warfarin [4]. The comparative effects of DOACs and VKAs may also vary across clinically distinct high-risk phenotypes, as demonstrated in evidence syntheses evaluating other underrepresented populations [37].

One potential effect modifier is the quality of baseline VKA anticoagulation. The clinical performance of VKA therapy depends heavily on anticoagulation control, and prior work has shown that time in therapeutic range below 70% is associated with higher risks of stroke/thromboembolism and major bleeding [38,39]. At the same time, high-quality control is not necessarily stable over time: in a Danish nationwide study, among AF patients with an initial TTR ≥ 70%, only about half remained above that threshold during follow-up [40]. Thus, the net effect of switching is likely to differ substantially between a frail elderly patient with persistently excellent INR control and one with labile anticoagulation. Because such information was incompletely available across the included studies, our pooled estimate should be interpreted as an average effect across patients with potentially very different baseline VKA quality.

Importantly, poor pre-switch VKA control does not automatically imply that a patient will do better after transition to a DOAC. In non-valvular AF patients switching from VKA to DOAC, Toorop et al. found that a low pre-switch TTR was associated with worse persistence after switching, suggesting that poor warfarin control may in some cases reflect broader treatment complexity rather than a problem solved simply by changing agents [41]. Conversely, Pundi et al. reported that most patients switched from warfarin to DOAC achieved adequate adherence, and that low pre-switch TTR should not be used as a direct surrogate for future DOAC nonadherence [42]. Analyzed together, these studies suggest that low TTR may capture a mixture of biological instability, comorbidity burden, healthcare fragmentation, and adherence challenges, all of which may continue to shape outcomes after switching.

A further hypothesis is that the period immediately after switching may represent a transient phase of increased vulnerability; however, this mechanism cannot be established from the present meta-analysis. A propensity-matched cohort study comparing short-term bleeding and arterial thromboembolism after oral anticoagulant initiation suggested that caution is warranted during early treatment periods, regardless of agent class [43]. In frail elderly patients, this early hazard may be amplified by fluctuating renal function, dose misalignment, polypharmacy, temporary overlap or mistiming during conversion, and heightened susceptibility to bleeding. Therefore, even if the long-term comparative efficacy of a DOAC is favorable in some settings, an early transition-related risk may attenuate or negate that benefit in frail patients already stable on warfarin.

Another key issue is that DOACs should not be treated as a fully uniform class in this context. Large real-world comparative studies in AF have consistently suggested clinically relevant differences between specific DOACs, with apixaban generally associated with lower major bleeding risk than rivaroxaban and, in some analyses, lower risks of both stroke and major bleeding relative to other agents [44,45,46]. Importantly, agent-specific differences were also evident within the studies included in the present meta-analysis. In the study by Kim et al. [12], switching from warfarin to apixaban was associated with a lower risk of the composite outcome, whereas switching to rivaroxaban was associated with a higher risk, largely driven by bleeding. Similarly, sensitivity analyses from COMBINE-AF [10] showed more favorable bleeding outcomes when the analysis was restricted to apixaban and edoxaban, while differences in the relative use of individual DOACs were proposed as a potential explanation for the divergent findings compared with FRAIL-AF [22]. This agent-level heterogeneity is especially relevant in frail elderly patients, in whom gastrointestinal vulnerability, renal impairment, low body weight, and concomitant medications may magnify pharmacologic differences. Accordingly, a pooled “DOAC versus VKA” analysis may obscure important within-class variation and partly explain why class-level neutrality can coexist with divergent signals for individual agents.

The present results therefore could support a more selective and clinically nuanced approach to switching [47]. They do not challenge the general preference for DOACs in most patients with AF; rather, they indicate that frail elderly patients who are already established on VKA therapy represent a distinct subgroup in whom the balance between benefit and harm may differ materially from the general AF population. In practical terms, patients with persistently poor TTR, unstable INR, or substantial burden from monitoring may still derive benefit from switching, whereas those with satisfactory anticoagulation control, advanced frailty, polypharmacy, gastrointestinal bleeding susceptibility, or uncertain adherence may not realize a net advantage. This interpretation aligns with contemporary guideline thinking that stable therapeutic VKA control remains a relevant modifier of treatment choice in older adults.

Finally, our findings highlight an important research gap. Future studies should move beyond broad class-level comparisons and instead identify the phenotypes most likely to benefit from switching. Such work should incorporate measured INR-based VKA quality, reasons for switching, agent-specific and dose-specific strategies, early post-switch event windows, and frailty characterization beyond administrative surrogates. Until such data are available, anticoagulant transition in frail elderly patients with AF should remain individualized rather than systematic.

### Strengths and Limitations

The present study has important strengths. It is, to our knowledge, the most comprehensive meta-analysis to date specifically addressing the question of switching from VKA to DOAC therapy in frail elderly patients with AF already maintained on long-term VKA treatment. Methodologically, it was based on a prespecified protocol, a broad systematic search strategy, and formal study-quality assessment, while also incorporating both randomized and real-world comparative data. The directional consistency between the primary analysis and the RCT-restricted sensitivity analysis suggests a broadly similar pattern of findings; however, the RCT-only analysis was based on only two comparisons and remained substantially heterogeneous and statistically imprecise for several outcomes.

Nevertheless, several limitations merit consideration. First, this was a study-level meta-analysis and therefore could not account for individual-patient factors that may have influenced treatment effect, including frailty burden, renal function, concomitant therapies, and dosing patterns. Second, substantial statistical heterogeneity was observed across most pooled outcomes. This likely reflects important clinical and methodological differences among the included studies, including the use of different frailty definitions and instruments, variation in study design, geographic setting, baseline patient characteristics and comorbidity burden, duration and stability of prior VKA treatment, and differences in the individual DOACs used. Importantly, frailty was not assessed uniformly across the included studies. The Groningen Frailty Indicator, FI-18, claims-based frailty index, and Hospital Frailty Risk Score capture different dimensions of vulnerability and rely on distinct clinical or administrative constructs. These instruments may therefore identify overlapping but clinically non-equivalent frail populations with different comorbidity burden, functional impairment, and baseline risk. Given that frailty defines the target population of this meta-analysis, such differences may have materially contributed to the observed between-study heterogeneity and should be considered when interpreting the pooled estimates and their generalizability. Given the small number of available studies, formal meta-regression or subgroup analyses exploring these sources of heterogeneity would not have been statistically reliable. Third, detailed information on the quality of VKA management was largely unavailable, particularly INR-based indices such as time in therapeutic range, which represents a major limitation given that the comparative value of switching may depend importantly on how well warfarin therapy was controlled before transition. Consequently, the pooled estimates may combine patients with substantially different baseline anticoagulation quality, potentially modifying the observed treatment effects and contributing to between-study heterogeneity. Fourth, the observational evidence remained susceptible to residual confounding and confounding by indication, because the rationale for switching was not captured and may have been influenced by unmeasured clinical factors. Fifth, some studies relied on administrative claims data, which limited ascertainment of clinically relevant non-major bleeding, laboratory-based bleeding definitions, cause-specific mortality, and other clinically nuanced outcomes. Finally, insufficiently granular data on specific DOAC agents and off-label dosing precluded more detailed treatment-stratified analyses. Accordingly, the findings should be interpreted as the best available comparative synthesis rather than as definitive evidence for all frail elderly patients receiving chronic VKA therapy.

## 5. Conclusions

In frail elderly patients with AF established on long-term VKA therapy, switching to a DOAC was not associated with statistically significant differences in composite outcome, major bleeding, thromboembolism, or all-cause mortality compared with continued VKA treatment. Given the limited number of studies, substantial heterogeneity, and imprecision of the pooled estimates, these findings should not be considered as evidence of equivalence between the two strategies. Clinically meaningful benefit or harm from switching cannot be excluded. Decisions regarding anticoagulant transition should therefore be individualized, and further prospective studies are needed to clarify the role of INR control, DOAC subtype, dosing, and patient selection in shaping outcomes after switching.

## Figures and Tables

**Table 1:** Characteristics of included studies.

Author, Year	Country/Design	Population/Frailty Definition	Follow-Up	DOAC Group, n	VKA Group, n	Age, Years	Female, %	CHA_2_DS_2_-VASc	Heart Failure, %	Hypertension, %	Diabetes, %	Prior Bleeding, %	Prior Thromboembolism/Stroke-TIA, %
Joosten LPT, 2024 [22]	Netherlands; pragmatic multicenter open-label RCT	AF, age ≥ 75 years, Groningen Frailty Indicator ≥ 3	12 months	662	661	DOAC: 83.0 ± 5.1; VKA: 82.8 ± 5.1	DOAC: 41.4; VKA: 36.2	DOAC: 4.0 (3.0–5.0); VKA: 4.0 (3.0–5.0)	DOAC: 19.5; VKA: 22.7	DOAC: 55.1; VKA: 50.8	DOAC: 21.1; VKA: 21.2	Major bleeding: DOAC 15.9; VKA 13.3	Thromboembolic history: DOAC 21.0; VKA 17.7
Nicolau AM, 2025 [10]	International; RCT	Frail, elderly (≥75 years), VKA-experienced AF; frailty defined as FI-18 ≥6	26.7 months	3005	2908	DOAC: 79.8 ± 3.7; VKA: 79.7 ± 3.7	DOAC: 41.2; VKA: 41.6	DOAC: 5 (4–6); VKA: 5 (4–6)	DOAC: 52.0; VKA: 50.3	DOAC: 92.4; VKA: 93.4	DOAC: 37.0; VKA: 37.8	GI bleeding history: DOAC 7.5, VKA 7.5; non-GI bleeding history: DOAC 14.5, VKA 14.1	Prior stroke/TIA: DOAC 31.6; VKA 32.9
Kim DH, 2025 (apixaban cohort) [12]	United States; retrospective nationwide multidatabase study, propensity score matched	AF receiving warfarin for ≥180 days; switching to apixaban vs. continuing warfarin; claims-based frailty index evaluated in subgroup analyses	12 months	11,429	11,429	DOAC: 80.14 ± 7.56; VKA: 80.15 ± 7.51	DOAC: 54.3; VKA: 54.7	DOAC: 3.99 ± 1.37; VKA: 3.99 ± 1.37	DOAC: 30.5; VKA: 30.4	DOAC: 70.2; VKA: 70.2	DOAC: 35.1; VKA: 35.0	HAS-BLED: DOAC 1.96 ± 0.69; VKA 1.97 ± 0.69	Prior stroke: DOAC 4.2; VKA 4.1
Kim DH, 2025 (rivaroxaban cohort) [12]	United States; retrospective nationwide multidata base study, propensity score matched	AF receiving warfarin for ≥180 days; switching to rivaroxaban vs. continuing warfarin; claims-based frailty index evaluated in subgroup analyses	12 months	7272	7272	DOAC: 79.32 ± 7.66; VKA: 79.33 ± 7.64	DOAC: 54.3; VKA: 54.5	DOAC: 3.91 ± 1.39; VKA: 3.90 ± 1.38	DOAC: 28.6; VKA: 28.3	DOAC: 69.4; VKA: 69.6	DOAC: 35.9; VKA: 35.5	HAS-BLED: DOAC 1.95 ± 0.68; VKA 1.95 ± 0.68	Prior stroke: DOAC 3.3; VKA 3.3
Lee SR, 2026 [11]	Korea; nationwide retrospective cohort with time-varying exposure analysis	Frail elderly Asian AF patients, age ≥ 75 years, Hospital Frailty Risk Score ≥ 5, stable on warfarin without major bleeding or thromboembolic events during screening	12 months	3349	9112	DOAC: 80.1 ± 4.1; VKA 80.5 ± 4.3	DOAC: 51.7; VKA: 49.7 *	DOAC: 5.1 ± 1.4; VKA: 5.0 ± 1.4	DOAC: 17.6; VKA: 11.7	DOAC: 89.3; VKA: 88.2	DOAC: 26.8; VKA: 28.8	Prior major bleeding: DOAC 13.3; VKA 10.5	Prior thromboembolic events: DOAC 33.6; VKA 31.8

Abbreviations: AF, atrial fibrillation; DOAC, direct oral anticoagulant; FI, frailty index; NR, not reported in the extracted table excerpt; RCT, randomized controlled trial; TIA, transient ischemic attack; VKA, vitamin K antagonist. Kim et al. [12] contributed two separate treatment comparisons (apixaban and rivaroxaban).

**Table 2:** GRADE Summary of Findings.

Outcomes	No of Participants (Studies)	Certainty of the Evidence (GRADE)	Relative Effect (95% CI)	Anticipated Absolute Effects	
				Risk with VKAs	Risk Difference with DOAC
Composite outcome	57,099 (4)	⨁⨁◯◯ Low ^a,b^	**HR 1.18** (0.84 to 1.67)	146 per 1.000	**24 more per 1.000** (from 22 fewer to 86 more)
Major bleeding	57,099 (4)	⨁⨁◯◯ Low ^a,b^	**HR 1.13** (0.80 to 1.60)	32 per 1.000	**4 more per 1.000** (from 6 fewer to 19 more)
Thromboembolism	57,099 (4)	⨁⨁◯◯ Low ^a,b^	**HR 1.03** (0.63 to 1.68)	18 per 1.000	**1 more per 1.000** (from 7 fewer to 12 more)
All-cause mortality	57,099 (4)	⨁⨁◯◯ Low ^a,b^	**HR 0.95** (0.77 to 1.16)	133 per 1.000	**6 fewer per 1.000** (from 29 fewer to 20 more)
* **The risk in the intervention group** (and its 95% confidence interval) is based on the assumed risk in the comparison group and the **relative effect** of the intervention (and its 95% CI). **CI:** confidence interval; **HR:** hazard ratio.					
**GRADE Working Group grades of evidence.** **High certainty:** we are very confident that the true effect lies close to that of the estimate of the effect. **Moderate certainty:** we are moderately confident in the effect estimate: the true effect is likely to be close to the estimate of the effect, but there is a possibility that it is substantially different. **Low certainty:** our confidence in the effect estimate is limited: the true effect may be substantially different from the estimate of the effect. **Very low certainty:** we have very little confidence in the effect estimate: the true effect is likely to be substantially different from the estimate of effect.					

Explanations: a. Downgraded one level for serious inconsistency due to substantial heterogeneity across studies and variability in the magnitude and direction of effect estimates. b. Downgraded one level for serious imprecision because the 95% confidence interval was wide and included both potentially clinically important benefits and harm.

## Data Availability

The original contributions presented in this study are included in the article/Supplementary Material. Further inquiries can be directed to the corresponding author.

## Supplementary Materials

The following supporting information can be downloaded at: https://www.mdpi.com/article/10.3390/medsci14050516/s1, Table S1: Search strategy for MEDLINE (Pubmed). Table S2: Search strategy for Cochrane library. Table S3: Search strategy for Scopus. Figure S1: Risk-of-bias assessments of included studies. Table S4: PRISMA 2020 Checklist.

## References

[1] Brundel B.J.J.M., Ai X., Hills M.T., Kuipers M.F., Lip G.Y.H., de Groot N.M.S. (2022). Atrial Fibrillation. Nat. Rev. Dis. Prim..

[2] Van Gelder I.C., Rienstra M., Bunting K.V., Casado-Arroyo R., Caso V., Crijns H.J.G.M., De Potter T.J.R., Dwight J., Guasti L., Hanke T. (2024). 2024 ESC Guidelines for the Management of Atrial Fibrillation Developed in Collaboration with the European Association for Cardio-Thoracic Surgery (EACTS). Eur. Heart J..

[3] Joglar J.A., Chung M.K., Armbruster A.L., Benjamin E.J., Chyou J.Y., Cronin E.M., Deswal A., Eckhardt L.L., Goldberger Z.D., Gopinathannair R. (2024). 2023 ACC/AHA/ACCP/HRS Guideline for the Diagnosis and Management of Atrial Fibrillation: A Report of the American College of Cardiology/American Heart Association Joint Committee on Clinical Practice Guidelines. Circulation.

[4] Carnicelli A.P., Hong H., Connolly S.J., Eikelboom J., Giugliano R.P., Morrow D.A., Patel M.R., Wallentin L., Alexander J.H., Cecilia Bahit M. (2022). Direct Oral Anticoagulants Versus Warfarin in Patients with Atrial Fibrillation: Patient-Level Network Meta-Analyses of Randomized Clinical Trials with Interaction Testing by Age and Sex. Circulation.

[5] Kim D.H., Pawar A., Gagne J.J., Bessette L.G., Lee H., Glynn R.J., Schneeweiss S. (2021). Frailty and Clinical Outcomes of Direct Oral Anticoagulants Versus Warfarin in Older Adults with Atrial Fibrillation: A Cohort Study. Ann. Intern. Med..

[6] Bul M., Shaikh F., McDonagh J., Ferguson C. (2023). Frailty and Oral Anticoagulant Prescription in Adults with Atrial Fibrillation: A Systematic Review. Aging Med..

[7] Bo M., Grisoglio E., Brunetti E., Falcone Y., Marchionni N. (2017). Oral Anticoagulant Therapy for Older Patients with Atrial Fibrillation: A Review of Current Evidence. Eur. J. Intern. Med..

[8] Calsolaro V., Okoye C., Antognoli R., Dell’Agnello U., Calabrese A.M., Monzani F. (2021). Long-Term Effectiveness and Safety of Anticoagulation Therapy in Oldest Old, Frail People with Atrial Fibrillation. Eur. J. Intern. Med..

[9] Bo M., Marchionni N. (2020). Practical Use of Direct Oral Anti Coagulants (DOACs) in the Older Persons with Atrial Fibrillation. Eur. J. Intern. Med..

[10] Nicolau A.M., Giugliano R.P., Zimerman A., Afilalo J., Gencer B., Steffel J., Palazzolo M.G., Eikelboom J.W., Granger C.B., Patel M.R. (2025). Outcomes in Older Patients After Switching to a Newer Anticoagulant or Remaining on Warfarin: The COMBINE-AF Substudy. J. Am. Coll. Cardiol..

[11] Lee S.-R., Go Y.-H., Choi E.-K., Rha H.-W., Jeong M.-H., Choi J., Lee K.-Y., Ahn H.-J., Kwon S., Kim B. (2026). Switching from Warfarin to Direct Oral Anticoagulants in Frail Elderly Asian Patients with Atrial Fibrillation: A Korean Nationwide Study. Eur. Heart J..

[12] Kim D.H., Ko D., Singer D.E., Cervone A., Zhang Y., Chen Q., Lin K.J. (2025). Clinical Outcomes of Switching From Warfarin to Apixaban or Rivaroxaban in Patients with Atrial Fibrillation: A Nationwide Multidatabase Study. Circ. Cardiovasc. Qual. Outcomes.

[13] Cochrane Handbook for Systematic Reviews of Interventions. Cochrane Training. https://training.cochrane.org/handbook/current.

[14] Page M.J., McKenzie J.E., Bossuyt P.M., Boutron I., Hoffmann T.C., Mulrow C.D., Shamseer L., Tetzlaff J.M., Akl E.A., Brennan S.E. (2021). The PRISMA 2020 Statement: An Updated Guideline for Reporting Systematic Reviews. BMJ.

[15] Haddaway N.R., Grainger M.J., Gray C.T. (2021). Citationchaser: An R Package and Shiny App for Forward and Backward Citations Chasing in Academic Searching, version 0.0.3.

[16] Wallace B.C., Small K., Brodley C.E., Lau J., Trikalinos T.A. (2012). Deploying an Interactive Machine Learning System in an Evidence-Based Practice Center: Abstrackr. IHI ’12: Proceedings of the 2nd ACM SIGHIT International Health Informatics Symposium.

[17] Sterne J.A.C., Savović J., Page M.J., Elbers R.G., Blencowe N.S., Boutron I., Cates C.J., Cheng H.Y., Corbett M.S., Eldridge S.M. (2019). RoB 2: A Revised Tool for Assessing Risk of Bias in Randomised Trials. BMJ.

[18] Sterne J.A., Hernán M.A., Reeves B.C., Savović J., Berkman N.D., Viswanathan M., Henry D., Altman D.G., Ansari M.T., Boutron I. (2016). ROBINS-I: A Tool for Assessing Risk of Bias in Non-Randomised Studies of Interventions. BMJ.

[19] Watkins C., Bennett I. (2018). A Simple Method for Combining Binomial Counts or Proportions with Hazard Ratios for Evidence Synthesis of Time-to-Event Data. Res. Synth. Methods.

[20] Karakasis P., Pamporis K., Siontis K.C., Theofilis P., Samaras A., Patoulias D., Stachteas P., Karagiannidis E., Stavropoulos G., Tzikas A. (2025). Major Clinical Outcomes in Symptomatic vs. Asymptomatic Atrial Fibrillation: A Meta-Analysis. Eur. Heart J..

[21] Chapter 10: Analysing Data and Undertaking Meta-Analyses. Cochrane Training.

[22] Joosten L.P.T., van Doorn S., van de Ven P.M., Köhlen B.T.G., Nierman M.C., Koek H.L., Hemels M.E.W., Huisman M.V., Kruip M., Faber L.M. (2024). Safety of Switching From a Vitamin K Antagonist to a Non-Vitamin K Antagonist Oral Anticoagulant in Frail Older Patients with Atrial Fibrillation: Results of the FRAIL-AF Randomized Controlled Trial. Circulation.

[23] Jaspers Focks J., Brouwer M.A., Wojdyla D.M., Thomas L., Lopes R.D., Washam J.B., Lanas F., Xavier D., Husted S., Wallentin L. (2016). Polypharmacy and Effects of Apixaban versus Warfarin in Patients with Atrial Fibrillation: Post Hoc Analysis of the ARISTOTLE Trial. BMJ.

[24] Piccini J.P., Hellkamp A.S., Washam J.B., Becker R.C., Breithardt G., Berkowitz S.D., Halperin J.L., Hankey G.J., Hacke W., Mahaffey K.W. (2016). Polypharmacy and the Efficacy and Safety of Rivaroxaban Versus Warfarin in the Prevention of Stroke in Patients with Nonvalvular Atrial Fibrillation. Circulation.

[25] Wilkinson C., Wu J., Searle S.D., Todd O., Hall M., Kunadian V., Clegg A., Rockwood K., Gale C.P. (2020). Clinical Outcomes in Patients with Atrial Fibrillation and Frailty: Insights from the ENGAGE AF-TIMI 48 Trial. BMC Med..

[26] Bo M., Fumagalli S., Degli Esposti L., Perrone V., Dovizio M., Poli D., Marcucci R., Verdecchia P., Reboldi G., Lip G.Y.H. (2024). Anticoagulation in Atrial Fibrillation. A Large Real-World Update. Eur. J. Intern. Med..

[27] Hellfritzsch M., Wang S.V., Grove E.L., Gagne J.J., Hallas J., Pottegård A. (2020). Using the Case-Crossover Design to Assess Short-Term Risks of Bleeding and Arterial Thromboembolism After Switching Between Oral Anticoagulants in a Population-Based Cohort of Patients with Atrial Fibrillation. Am. J. Epidemiol..

[28] Sørensen R., Gislason G., Torp-Pedersen C., Olesen J.B., Fosbøl E.L., Hvidtfeldt M.W., Karasoy D., Lamberts M., Charlot M., Køber L. (2013). Dabigatran Use in Danish Atrial Fibrillation Patients in 2011: A Nationwide Study. BMJ Open.

[29] Norby F.L., Bengtson L.G.S., Lutsey P.L., Chen L.Y., MacLehose R.F., Chamberlain A.M., Rapson I., Alonso A. (2017). Comparative Effectiveness of Rivaroxaban versus Warfarin or Dabigatran for the Treatment of Patients with Non-Valvular Atrial Fibrillation. BMC Cardiovasc. Disord..

[30] Bengtson L.G.S., Lutsey P.L., Chen L.Y., MacLehose R.F., Alonso A. (2017). Comparative Effectiveness of Dabigatran and Rivaroxaban versus Warfarin for the Treatment of Non-Valvular Atrial Fibrillation. J. Cardiol..

[31] Bellin A., Berto P., Themistoclakis S., Chandak A., Giusti P., Cavalli G., Bakshi S., Tessarin M., Deambrosis P., Chinellato A. (2019). New Oral Anti-Coagulants versus Vitamin K Antagonists in High Thromboembolic Risk Patients. PLoS ONE.

[32] Bouillon K., Bertrand M., Maura G., Blotière P.-O., Ricordeau P., Zureik M. (2015). Risk of Bleeding and Arterial Thromboembolism in Patients with Non-Valvular Atrial Fibrillation Either Maintained on a Vitamin K Antagonist or Switched to a Non-Vitamin K-Antagonist Oral Anticoagulant: A Retrospective, Matched-Cohort Study. Lancet Haematol..

[33] Vaughan Sarrazin M.S., Jones M., Mazur A., Chrischilles E., Cram P. (2014). Bleeding Rates in Veterans Affairs Patients with Atrial Fibrillation Who Switch from Warfarin to Dabigatran. Am. J. Med..

[34] Larsen T.B., Gorst-Rasmussen A., Rasmussen L.H., Skjøth F., Rosenzweig M., Lip G.Y.H. (2014). Bleeding Events among New Starters and Switchers to Dabigatran Compared with Warfarin in Atrial Fibrillation. Am. J. Med..

[35] Larsen T.B., Rasmussen L.H., Gorst-Rasmussen A., Skjøth F., Lane D.A., Lip G.Y.H. (2014). Dabigatran and Warfarin for Secondary Prevention of Stroke in Atrial Fibrillation Patients: A Nationwide Cohort Study. Am. J. Med..

[36] Sciria C.T., Maddox T.M., Marzec L., Rodwin B., Virani S.S., Annapureddy A., Freeman J.V., O’Hare A., Liu Y., Song Y. (2020). Switching Warfarin to Direct Oral Anticoagulants in Atrial Fibrillation: Insights from the NCDR PINNACLE Registry. Clin. Cardiol..

[37] Karakasis P., Ktenopoulos N., Pamporis K., Sagris M., Soulaidopoulos S., Gerogianni M., Leontsinis I., Giannakoulas G., Tousoulis D., Fragakis N. (2024). Efficacy and Safety of Direct Oral Anticoagulants versus Warfarin in Obese Patients (BMI ≥ 30 Kg/m^2^) with Atrial Fibrillation or Venous Thromboembolism: An Updated Systematic Review and Meta-Analysis. J. Clin. Med..

[38] McAlister F.A., Wiebe N., Hemmelgarn B.R. (2018). Time in Therapeutic Range and Stability over Time for Warfarin Users in Clinical Practice: A Retrospective Cohort Study Using Linked Routinely Collected Health Data in Alberta, Canada. BMJ Open.

[39] Wan Y., Heneghan C., Perera R., Roberts N., Hollowell J., Glasziou P., Bankhead C., Xu Y. (2008). Anticoagulation Control and Prediction of Adverse Events in Patients with Atrial Fibrillation: A Systematic Review. Circ. Cardiovasc. Qual. Outcomes.

[40] Bonde A.N., Staerk L., Lee C.J.-Y., Vinding N.E., Bang C.N., Torp-Pedersen C., Gislason G., Lip G.Y.H., Olesen J.B. (2018). Outcomes Among Patients with Atrial Fibrillation and Appropriate Anticoagulation Control. J. Am. Coll. Cardiol..

[41] Toorop M.M.A., Chen Q., Kruip M.J.H.A., van der Meer F.J.M., Nierman M.C., Faber L., Goede L., Cannegieter S.C., Lijfering W.M. (2022). Switching from Vitamin K Antagonists to Direct Oral Anticoagulants in Non-Valvular Atrial Fibrillation Patients: Does Low Time in Therapeutic Range Affect Persistence?. J. Thromb. Haemost..

[42] Pundi K.N., Perino A.C., Fan J., Schmitt S., Kothari M., Szummer K., Askari M., Heidenreich P.A., Turakhia M.P. (2021). Direct Oral Anticoagulant Adherence of Patients with Atrial Fibrillation Transitioned from Warfarin. J. Am. Heart Assoc..

[43] Maura G., Blotière P.-O., Bouillon K., Billionnet C., Ricordeau P., Alla F., Zureik M. (2015). Comparison of the Short-Term Risk of Bleeding and Arterial Thromboembolic Events in Nonvalvular Atrial Fibrillation Patients Newly Treated with Dabigatran or Rivaroxaban versus Vitamin K Antagonists: A French Nationwide Propensity-Matched Cohort Study. Circulation.

[44] Chan Y.-H., See L.-C., Tu H.-T., Yeh Y.-H., Chang S.-H., Wu L.-S., Lee H.-F., Wang C.-L., Kuo C.-F., Kuo C.-T. (2018). Efficacy and Safety of Apixaban, Dabigatran, Rivaroxaban, and Warfarin in Asians with Nonvalvular Atrial Fibrillation. J. Am. Heart Assoc..

[45] Lamberts M., Staerk L., Olesen J.B., Fosbøl E.L., Hansen M.L., Harboe L., Lefevre C., Evans D., Gislason G.H. (2017). Major Bleeding Complications and Persistence with Oral Anticoagulation in Non-Valvular Atrial Fibrillation: Contemporary Findings in Real-Life Danish Patients. J. Am. Heart Assoc..

[46] Yao X., Abraham N.S., Sangaralingham L.R., Bellolio M.F., McBane R.D., Shah N.D., Noseworthy P.A. (2016). Effectiveness and Safety of Dabigatran, Rivaroxaban, and Apixaban Versus Warfarin in Nonvalvular Atrial Fibrillation. J. Am. Heart Assoc..

[47] Karakasis P. (2026). To Switch or Not to Switch: Rethinking Anticoagulation in Frail Elderly Patients. Eur. Heart J..

